# Bioinformatic-driven search for metabolic biomarkers in disease

**DOI:** 10.1186/2043-9113-1-2

**Published:** 2011-01-20

**Authors:** Christian Baumgartner, Melanie Osl, Michael Netzer, Daniela Baumgartner

**Affiliations:** 1 Research Group for Clinical Bioinformatics, Institute of Electrical, Electronic and Bioengineering, University for Health Sciences, Medical Informatics and Technology (UMIT), Hall in Tirol, Austria; 2 Clinical Division of Pediatric Cardiology, Department of Pediatrics, Innsbruck Medical University, Austria

## Abstract

The search and validation of novel disease biomarkers requires the complementary power of professional study planning and execution, modern profiling technologies and related bioinformatics tools for data analysis and interpretation. Biomarkers have considerable impact on the care of patients and are urgently needed for advancing diagnostics, prognostics and treatment of disease. This survey article highlights emerging bioinformatics methods for biomarker discovery in clinical metabolomics, focusing on the problem of data preprocessing and consolidation, the data-driven search, verification, prioritization and biological interpretation of putative metabolic candidate biomarkers in disease. In particular, data mining tools suitable for the application to omic data gathered from most frequently-used type of experimental designs, such as case-control or longitudinal biomarker cohort studies, are reviewed and case examples of selected discovery steps are delineated in more detail. This review demonstrates that clinical bioinformatics has evolved into an essential element of biomarker discovery, translating new innovations and successes in profiling technologies and bioinformatics to clinical application.

## Biomarkers, profiling technologies and bioinformatics

By definition, biomarkers are "objectively measured indicators of normal biological processes, pathogenic processes or pharmacological responses to a therapeutic intervention, and ... are intended to substitute for a clinical endpoint (predict benefit or harm) based on epidemiological, therapeutic, pathophysiological or other scientific evidence (Biomarkers Definitions Working Group, 2001)" and have a variety of functions [1]. From the clinical perspective, biomarkers have a substantial impact on the care of patients who are suspected to have disease, or those who have or have no apparent disease. According to this categorization, biomarkers can be classified into diagnostic, prognostic and screening biomarkers. The latter are of high interest because of their ability to predict future events, but currently there are few accepted biomarkers for disease screening [2-4].

Advances in omic profiling technologies allow the systemic analysis and characterization of alterations in genes, RNA, proteins and metabolites, and offer the possibility of discovering novel biomarkers and pathways activated in disease or associated with disease conditions [5-7]. The proteome, as an example, is highly dynamic due to the diversity and regulative structure of posttranslational modifications, and gives an in-depth insight into disease; this is because protein biomarkers reflect the state of a cell or cellular subsystem determined by expression of a set of common genes. Many interesting proteins related to human disease, however, are low-abundance molecules and can be analyzed by modern mass-spectrometry (MS) -based proteomics instrumentations, even if these technologies are somewhat limited due to their moderate sensitivity and the dynamic range necessary for high-throughput analysis [8]. In metabolomics, metabolite profiling platforms, using tandem mass spectrometry (MS/MS) coupled with liquid chromatography (LC), allow the analysis of low-molecular weight analytes in biological mixtures such as blood, urine or tissue with high sensitivity and structural specificity, but still preclude the analysis of large numbers of samples [9,10]. More recently, whole spectrum analysis of the human breath in liver disease or cancer using ion-molecule reaction (IMR) or proton transfer reaction (PTR) mass spectrometry represents a further layer of potential applications in the field of biomarker discovery, as a breath sample can be obtained non-invasively and its constituents directly reflect concentrations in the blood [11,12].

In general, the search, verification, biological and biochemical interpretation and independent validation of disease biomarkers require new innovations in high-throughput technologies, biostatistics and bioinformatics, and thus make necessary the interdisciplinary expertise and teamwork of clinicians, biologists, analytical- and biochemists, and bioinformaticians to carry out all steps of a biomarker cohort study with professional planning, implementation, and control. Generally in human biomarker discovery studies, a variety of experimental designs are used. These include case-control or more complex cohort study designs such as crossover or serial sampling designs. Retrospective case-control studies is the type of epidemiological study most frequently used to identify biomarkers, by comparing patients who have a specific medical condition (cases) with individuals who do not have this condition but have other similar phenotypic and patient specific characteristics (controls). In contrast, longitudinal cohort studies allow patients to serve as their own biological control, which reduces the interindividual variability observed in multiple cohort studies as well as the technology platform-based variability due to a moderate signal-to-noise ratio [13].

**Figure 1:**
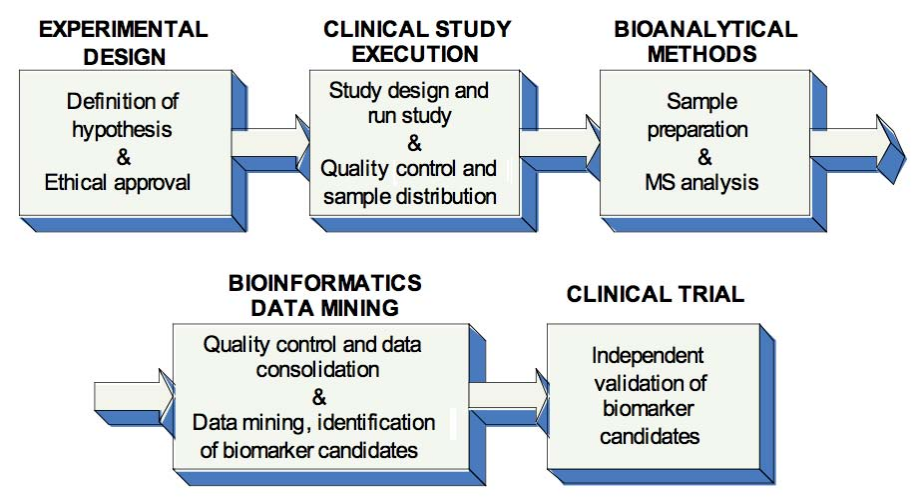
**Biomarker discovery process in human disease using an MS-based metabolite profiling platform**.

Bioinformatics plays a key role in the biomarker discovery process, bridging the gap between initial discovery phases such as experimental design, clinical study execution, and bioanalytics, including sample preparation, separation and high-throughput profiling and independent validation of identified candidate biomarkers. Figure. 1 shows the typical workflow of a biomarker discovery process in clinical metabolomics.

In this survey article, we review and discuss emerging bioinformatic approaches for metabolomic biomarker discovery in human disease, delineating how data mining concepts are being selected and applied to the problem of identifying, prioritizing, interpreting and validating clinically useful metabolic biomarkers.

## Quality controlled collection and integration of biomedical data

Central to biomedical research is a Good Clinical Practice (GCP) compliant data collection of patient-related records, which accommodates the quality controlled collection and tracking of samples and additional study material. This practice necessitates a carefully executed, standardized integration of generated omic/epigenetic data and clinical information including biochemistry, pathology and follow-up. If required, it also must be made complete with data from public repositories such as Enzyme, KEGG, Gene Ontology, NCBI Taxonomy, SwissProt or TrEMBL and literature (e.g PubMed) using appropriate data warehouse solutions. In the past few years in particular, the bioinformatics community has made great progress in developing data warehouse applications in a biomedical context for improved management and integration of the large volumes of data generated by various disciplines in life sciences.

A data warehouse is a central collection or repository that continuously and permanently stores all of the relevant data and information for analysis. Coupled with intelligent search, data mining and discovery tools, it enables the collection and processing of these data to turn them into new biomedical knowledge [14,15]. Technically, we need to distinguish between the back room and front room entities, as these two parts are usually separated physically and logically. While the back room holds and manages the data, the front room usually enables data accession and data mining. In comprehensive biomarker cohort studies, a data warehouse is an essential bioinformatic tool for standardized collection and integration of biomedical data, as well as meta-analysis of clinical, omic and literature data under the constraints of well-phenotyped patients' cohorts to discover and establish new biomarkers for early diagnosis and treatment.

## Fundamental statistic concepts, data mining methods and meta-analysis

Once a biomarker cohort study has been set up, and sample collection, preparation, separation and MS analysis have been carried out, an extensive technical review of generated data is essential to ensure a high degree of consistency, completeness and reproducibility in the data.

Data preprocessing, as a preliminary data mining practice performed on the raw data, is necessary to transform data into a format that will be more easily and effectively processed for the purpose of targeted analyses. There are a number of methods used for data preprocessing, including data transformation (e.g. logarithmic scaling of data) and normalization, e.g. using z-transformation, data sampling or outlier detection. In particular, the problem of detecting and cleaning datasets from outliers is a crucial task in data preprocessing. Thus, a careful handling of outliers is warranted to avoid manipulation and distortion of statistical results, which complicates a useful interpretation of biological findings. Traditional statistical approaches propose observations as outliers that are deemed unlikely with respect to mean and standard deviation, assuming normal data distribution. A common model uses the interquartile ranges and defines an outlier as observation outside the interquartile range IQR = Q_3 _- Q_1_, where Q_1 _and Q_3 _are the first and third quartiles. However, alternative data mining methods try to overcome concepts based on the assumption that data is normally distributed, by using distance-based approaches or defining the outlier problem via a local neighborhood of data points in a given data space, such as the local outlier factor (LOF) or the algorithm LOCI, using a local correlation integral for detecting outliers [16-18]. These methods show high value in treating the problem of outlier detection, especially in multiple biomarker search problems.

In recent years, various powerful data mining and statistical bioinformatics methods have been propagated for identifying, prioritizing and classifying robust and generalizable biomarkers with high discriminatory ability [19-27]. Principal data mining tasks in biomarker discovery, such as the identification of biomarker candidates in experimental data (feature selection) and classification, are "supervised" because study cohorts are well phenotyped in carefully designed and controlled clinical trials. Therefore, data vectors are determined by a set of tuples, *T = {(c*_*j*_*, a) | c*_*j*_*∈ C, a ∈ A}*, where *c*_*j *_is a class label from the collection *C *of pre-classified cohorts (normal, diseased, various stages of disease, treated, at rest, during stress, etc.), and *A = {a | a*_*1*_*, ... , a*_*n*_*} *is the set of concentrations of low-molecular weight biomolecules such as nucleotides, amino and organic acids, lipids, sugars, etc., if molecules are predefined and quantified, or simple m/z values from generated raw mass spectra. In this area, basic data mining concepts for the search of biomarker candidates constitute filter- and wrapper-based feature selection algorithms, and more advanced paradigms like embedded or ensemble methods [27-31]. However, if class membership is (partly) unknown, semi- or unsupervised techniques (cluster analysis) are helpful tools for biomarker search and interpretation. Note that many unsupervised feature selection methods treat this task as a search problem. Since the data space is exponential in the number of examined features, the use of heuristic search procedures are necessary where the search is combined with a feature utility estimator to evaluate and assess the relative merit of selected subsets of features. Supervised clustering, for example, opens a new research field in biomarker discovery to be employed when class labels of all data are known, with the objective of finding class pure clusters. Table 1 gives a survey of widely-used supervised feature selection techniques, useful for the identification of candidate biomarkers in data sets gathered from well-phenotyped cohort studies, considering both basic types of paired and unpaired test hypotheses [32-40].

**Table 1:** Commonly used supervised data mining methods for the search and prioritization of biomarker candidates in independent and dependent samples

Independent samples	Method	Basic principle and key features of the method	Reference
	Unpaired null hypothesis testing (Two-sample t-test*, Mann-Whitney-U test°)	- univariate filter method - P value serves as evaluation measure for the discriminatory ability of variables - is an accepted statistical measure - appropriate for two class problems only - P value is sample size dependent	Lehmann, *Springer Verlag*, 2005 [32]
	
	Principal component analysis (PCA)^#^	- unsupervised projection method - PCA calculates linear combinations of variables based on the variance of the original data space - appropriate for multiple class problems - visualizable loading and score plots (scores can be labeled according to class membership) - no ranking and prioritization of features possible	Jolliffe, *Springer Verlag*, 2005 [33], Ringnér, *Nat Biotechnol*, 2008 [34]
	
	Information gain (IG)	- univariate filter method - IG calculates how well a given feature separates data by pursuing reduction of entropy - appropriate for multiple class problems - quick and effective ranking of features - IG scores permit prioritization of features	Hall and Holmes, *IEEE Trans Knowl Data Eng*, 2003 [28]
	
	ReliefF (RF)	- multivariate filter method - RF score relies on the concept that values of a significant feature are correlated with the feature values of an instance of the same class, and uncorrelated with the feature values of an instance of the other class - appropriate for multiple class problems - RF scores permit prioritization of features	Robnik-Sikonja & Kononenko, *Mach Learn*, 2003 [35] Hall and Holmes, *IEEE Trans Knowl Data Eng*, 2003 [28]
	
	Associative voting (AV)	- multivariate filter method - AV uses a rule-based evaluation criterion by a special form of association rules; considers interaction among features - appropriate for two class problems only - AV scores permit prioritization of features - restriction of the rule search space necessary	Osl et al., *Bioinformatics*, 2008 [36]
	
	Unpaired Biomarker Identifier (uBI)	- univariate filter method - statistical evaluation score by combining a discriminance measure with a biological effect term - appropriate for two class problems only - quick and effective ranking of features - uBI scores permit prioritization of features - uBI scores closely related to pBI scores	Baumgartner et al., *Bioinformatics*, 2010 [13]
	
	Guilt-by-association feature selection (GBA-FS)	- multivariate subset selection method - GBA-FS uses a hierarchical clustering with correlation as distance measure; the most relevant features of each cluster are assessed by their discriminatory power, as measured for example by two-sample t-test - accounts for redundancy between features - appropriate for two class problems only	Shin et al., *J Biomed Inform*, 2007 [37]
	
	Support vector machine-recursive feature elimination (SVM-REF)	- embedded selection method - SVM-REF uses optimized weights of SVM classifier to rank features - appropriate for two class problems only	Guyon et al., *Mach Learn*, 2002 [38]
	
	Random forest models (RFM)	- embedded selection method - RFM uses bagging and random subspace methods to construct a collection of decision trees aiming at identifying a complete set of significant features - appropriate for multiple class problems	Enot et al., *PNAS*, 2006 [39]
	
	Aggregating feature selection (AFS)	- ensemble selection method - aggregating multiple feature selection results to a consensus ranking, e.g. using the concept of weighted voting or by counting the most frequently selected features to derive the consensus feature subset - appropriate for multiple class problems	Saeys et al., *Lecture Notes in Artificial Intelligence*, 2008 [30]
	
	Stacked feature ranking (SFR)	- ensemble selection method - stacked learning architecture to construct a consensus feature ranking by combining multiple feature selection methods - appropriate for multiple class problems - feature selection by optimizing the discriminatory ability (AUC)	Netzer et al., *Bioinformatics*, 2009 [31]
	
	Wrapper approach	- evaluating the merit of a feature subset by accuracy estimates using a classifier - produces subsets of very few features that are dominated by stronger and uncorrelated attributes - increased computational runtime; necessitates heuristic search methods like forward selection, backward elimination, or more sophisticated methods such as genetic algorithms	Hall and Holmes, *IEEE Trans Knowl Data Eng*, 2003 [28]

**Dependent samples**	Paired null hypothesis testing (Paired t-test*, Wilcoxon signed-rank test°)	- univariate filter method - P value serves as evaluation measure for the discriminatory ability of variables - is an accepted statistical measure - appropriate for two class problems only - P value is sample size dependent - two dependent samples	Lehmann, *Springer Verlag*, 2005 [32]
	
	Repeated measure analysis	- univariate and multivariate approaches - mixed model analysis (GLMM, General Linear Mixed Model) - time series (multiple time points) analysis	Crowder & Hand, *Analysis of repeated measures*, 1990 [40]
	
	Paired Biomarker Identifier (pBI)	- univariate filter method - pBI uses a statistical evaluation score by combining a discriminance measure with a biological effect term - appropriate for two class problems only - pBI scores permit prioritization of features - pBI scores closely related to uBI scores	Baumgartner et al., *Bioinformatics*, 2010 [13]

* data normal distributed, ° data non-normal distributed. ^# ^PCA is an unsupervised method also used for data containing class information. All algorithms are run on continuous data as data generated in metabolomics are usually of metric nature. Data can represent absolute metabolite concentrations (given as intensity counts or more specific in μmol/L if internal standards are available) or simple m/z values from raw or preprocessed mass spectra.

Recently, combined biomarkers constructed by mathematical expressions such as quotients or products have been utilized to significantly enhance their predictive value, as demonstrated in newborn screening [41,42]. For example, a simple model for screening for phenylananine hydroxylase deficiency (PKU), a common congenital error of metabolism, was proposed by the ratio Phe/Tyr (Phe is phenylananine and Thy is tyrosine), to describe the irreversible reaction A→B of a reactant A into a product B, caused by an impaired enzyme activity [43]. In this manner, models of single and combined predictors, as built upon *a priori *knowledge of abnormal pathways like those shown above, exhibit high potential to develop screening models with high discriminatory ability. Ultimately, the process of identifying clinically relevant biomarkers is an ambitious data-mining task, bringing together various computational concepts of feature ranking, subset selection and feature construction by attribute combination.

The identification of a set of relevant, but not redundant, predictors is important for building prognostic and diagnostic models. Ding and Peng, for example, presented a minimum redundancy feature selection approach on microarray data, demonstrating significantly better classification accuracy on selected minimized redundant gene sets than those obtained through standard feature ranking methods [44]. Most commonly, individual features are ranked in terms of a quality criterion, out of which the top *k *features are selected. However, most feature-ranking methods do not sufficiently account for interactions and correlations between the features, and therefore redundancy is likely to be encountered in the selected features. Recently, Osl et al., presented a new algorithm, termed Redundancy Demoting (RD), that takes an arbitrary feature ranking as input, and improves the predictive value of a selected feature subset by identifying and demoting redundant features in a postprocessing modality [45]. The authors define redundant features as those that are correlated with other features, but are not relevant in the sense that they do not improve the discriminatory ability of a selected feature set. This means that although correlated biomarkers exhibit potential reactions and interactions among biomolecules in a biological pathway, they do not provide a substantial increase in predictive value if they are redundant. On the other hand, if they are not redundant, they may be good candidates to further enhance the predictive value of selected multiple biomarkers.

For building predictive models on biological data, a wide spectrum of machine learning methods is available: These include discriminant analysis methods like linear discriminant analysis or logistic regression analysis, decision trees, the k-nearest neighbor classifier (k-NN), an instance-based learning algorithm, the Bayes classifier, a probabilistic method based on applying the Bayes' theorem, support vector machines, a method that uses a kernel technique to apply linear classification techniques to nonlinear classification problems or artificial neural networks [46-53]. A more detailed review of these methods, however, is beyond the scope of this article.

As an advanced and more sophisticated layer of data analysis, meta-analysis is used with the objective of improving single experiment results and identifying common clinical and biological relevant patterns [54,55]. Meta-analysis of data may contain different steps: (i) scoring disease-relevance of candidate biomarkers by integrated analysis of the different clinical and experimental data (which may arise from multiple clinical studies), (ii) building statistical models on preselected candidates, derived by coupling methods such as feature selection and logistic regression analysis that result in the highest discriminatory ability with respect to the targeted patient cohorts or populations, (iii) performing correlation analysis to analyze 'omics' data under constraints defined by the patient data, (vi) examining various performance characteristics of biomarker candidates e.g. through decision-analytic outcome modeling. Receiver-operating-characteristics (ROC) analyses of related discriminatory models with specific sensitivities and specificities are used as input parameters for decision models, calculating expected epidemiologic and economic consequences for individuals and public health of the evolving health-care technologies under assessment.

## Generalizability and validation of biomarkers

Objective measures to assess the predictive value and generalizable power of selected candidate biomarkers are sensitivity, specificity, the product of sensitivity and specificity, or the area under the ROC curve (AUC). These measures are useful and valid only if they are determined on independent samples (e.g. cases versus controls). In serial sampling studies, alternative measures are needed to assess the predictive value of biomarkers in a similar manner. Very recently, a new objective measure for expressing the discriminatory ability (DA) in dependent samples was developed by our group [13]. The discriminance measure DA is defined as the percent change of analyte levels in a cohort in one direction versus baseline, and acts as a feature analogously to the product of sensitivity and specificity when addressing an unpaired test problem. Thus, a DA value of 0.5 in paired testing corresponds exactly to a product or AUC of 0.5 in unpaired testing, demonstrating no discrimination, while a DA of 0.75 or 1.00 indicates good or perfect discrimination.

**Figure 2:**
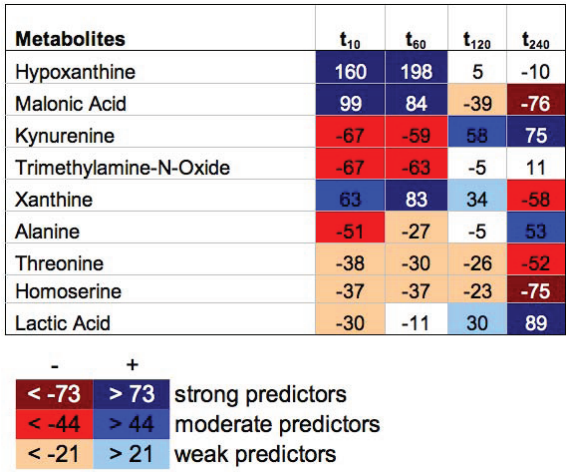
**Kinetic map of metabolites on PMI data at 10, 60, 120, and 240 minutes after myocardial injury, using the pBI scoring model for prioritization of selected metabolites into groups of weak, moderate and strong predictors**. Values indicate absolute pBI scores. The thresholds for prioritization are denoted below in the list of analytes. Red color increments indicate decreasing levels, blue increasing levels. In this study, a series of metabolites in pathways associated with myocardial infarction could be identified, some of which change as early as 10 minutes after injury, a time frame where no currently available clinical biomarkers are present [13,56].

Using both related discrimination measures, i.e. the product of sensitivity and specificity, and DA, a clinically useful prioritization of biomarkers - for example, into classes of weak, moderate and strong predictors - is possible independently of the study design (e.g. case-control versus serial sampling study). Very recently, Lewis et al. and Baumgartner et al. published a prospective longitudinal biomarker cohort study that was carried out to identify, categorize, and profile kinetic patterns of early metabolic biomarkers of planned (PMI) and spontaneous (SMI) myocardial infarction [56,13]. Figure. 2 depicts a kinetic map of selected circulating metabolites from a human model of PMI that faithfully reproduces SMI [57]. Promising metabolites were selected and prioritized into classes of different predictive value by using the so-called pBI scoring model, developed for longitudinal biomarker cohort studies where each patient serves as his/her own control [13]. In the given example, each circulating metabolite is able to be categorized at each time point of analysis in order to qualitatively and quantitatively assess the dynamic expression pattern of metabolic biomarkers after myocardial injury. Using this approach, a set of promising putative biomarker candidates could be identified as early as 10 minutes after the event.

In general, identified biomarker candidates need to be validated using larger sample sets, covering a broad cross section of patients or populations. However, if no independent cohort for validation is available, especially if further samples are costly, hazardous or impossible to collect, cross validation is an accepted statistical strategy to assess generalizability on a single derivation cohort at this discovery stage. Usually, stratified 10-fold cross-validation is applied, which is the statistical practice of partitioning a sample of data into ten subsets, where each subset is used for testing and the remainder for training, yielding an averaged overall error estimate. For very small samples, leave-one-out cross validation using one observation for testing and n-1 observations for training is proposed to generalize findings. Alternatively, bootstrapping or permutation modalities can be used as powerful approaches for statistical validation [58-60].

**Figure 3:**
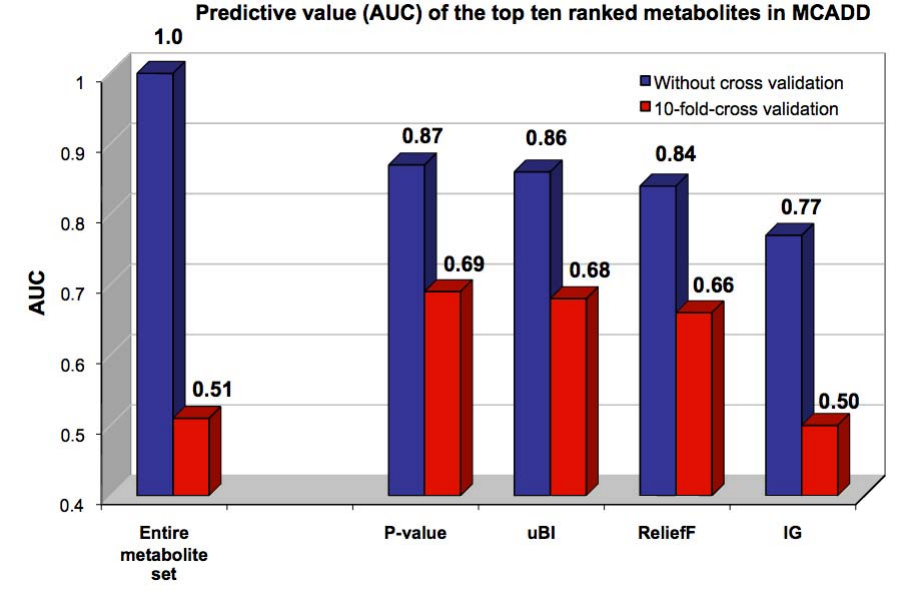
**AUC analysis on the entire metabolite set (bars in the left), and on a set of the top ten ranked metabolites using four common feature selection methods, i.e. two sample t-test (P-value), the unpaired Biomarker Identifier (uBI), ReliefF, and Information gain (IG) on MCADD data (bars in the right)**. Red bars represent the predictive value expressed by the AUC of selected analyte sets, determined on a single derivation cohort *with *cross validation and blue bars *without *cross-validation. Interestingly, using the entire metabolite set (43 analytes) for distinguishing between the two groups, the discriminatory ability dropped from AUC = 1.0 (without cross validation) to AUC = 0.51 after 10-fold cross validation, thus indicating no discrimination between the cohorts. On the selected subset, the AUC dropped by 15% to 25% after cross validation, demonstrating weak predictive value and thus low generalizability of the selected subset in this experiment.

As an example, Figure. 3 shows the predictive value of multiple metabolites in newborn screening data on a single derivation cohort *with *and *without *stratified 10-fold cross validation. The data set contains concentrations of 43 analytes, i.e. amino acids and acyl-carnitines, separated into 63 cases (medium-chain acyl-CoA dehydrogenase deficiency, MCADD) and 1241 healthy controls [61]. This result clearly demonstrates the strong disagreement in discriminatory ability between non- and cross-validated analyte subsets, and confirms the necessity of this computational modality for pre-selecting robust and generalizable candidate biomarkers, eliminating the potential bottleneck of taking too many candidates to the validation phase. Meta-analysis is a next logical step to further strengthen such results. However, after these crucial discovery steps, prospective trials are ultimately needed to validate the clinical benefit of assessing expression patterns of selected biomarker candidates before they can go into clinical routine.

## Analysis after biomarker identification

**Figure 4:**
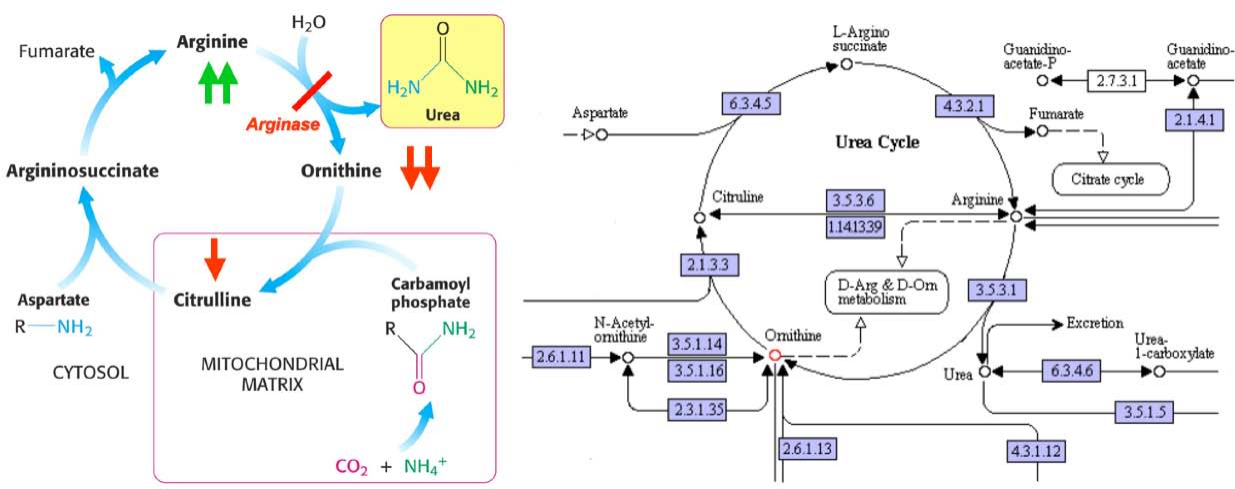
**The high and low concentration levels of arginine (Arg) and ornithine (Orn), respectively, in patients afflicted with severe metabolic syndrome and cardiovascular disease (MS+) versus healthy controls, implied an impacted enzyme arginase in the urea cycle (left figure)**. The urea cycle and associated pathways from the KEGG database are depicted in the right figure. Findings could be confirmed by literature [66,67].

One challenging research area in bioinformatics is the biological and biochemical interpretation of identified putative marker candidates by means of mining the most likely pathways. In metabolomics, various explorer tools such as cPath, Pathway Hunter Tool (public) or Ingenuity Pathway Analysis and MetaCore (commercial) are available to visualize, map and reconstruct a spectrum of possible pathways between relevant metabolites identified by feature selection [62,63]. Most tools extract metabolic information from metabolic network databases like KEGG and provide algorithms which allow (i) querying of thousands of endogenous analytes from those databases, (ii) displaying biochemical pathways with their involved metabolite and enzymes, and (iii) reconstructing and visualizing the most likely pathways related to the identified key metabolites [24,64,65]. These tools also provide an interactive analysis of biochemical pathways and entities such as metabolites, enzymes or reactions and allow a quick and direct functional annotation of experimental findings. As an example, Figure. 4 shows the most likely pathway in the KEGG database, addressing altered concentration levels of arginine (Arg) and ornithine (Orn), respectively, in patients afflicted with severe metabolic syndrome and cardiovascular disease (MS+) versus healthy controls. Both candidate metabolites, which are closely associated with the D-Arg & D-Orn metabolism in the urea cycle, were identified by feature selection from targeted MS profiling data [24,66,67].

Direct hyperlinks to databases such as OMIM, Swiss-Prot or Prosite reveal supplementary information about these entities that can help researchers learn more about the underlying biochemical and biological mechanisms. It is obvious that emerging bioinformatics tools for exploring metabolic pathways and networks, thus allowing for mapping expression profiles of genes or proteins simultaneously onto these pathways, are of high importance for the biological interpretation of biomarkers from a systems biology viewpoint [68-70]. Such tools thus contribute to a better understanding of how genes, proteins and metabolites act and interact in such networks, and consequently how human diseases manifest themselves.

## Conclusions and final remarks

In this article we have discussed the complementary power of modern profiling technologies and bioinformatics for metabolomic biomarker discovery in human disease. The discovery and interpretation of new biomarkers, however, depends on a comprehensive view of genomics, transcriptomics, proteomics and metabolomics [71]. In particular, proteomics and metabolomics offer excellent insights into disease, because function, structure or turnover of proteins, typically regulated via post-translational modifications, as well as metabolites, which act as end products of cellular processes, define the phenotypic heterogeneity of disease [72-74]. Therefore, great interest in the discovery of new biomarkers originates from their wide range of clinical applications, fundamental impact on pharmaceutical industry, and the current public health burden. Biomarkers, once qualified for clinical use, can aid in diagnosis and prediction of life-threatening events, confirm drug's pharmacological or biological action mechanisms, or serve as early and objective indicators of treatment efficiency in patients [75-78]. Theranostics, an emerging field in personalized medicine, utilizes molecular biomarkers to select patients for treatments that are expected to benefit them and are unlikely to produce side effects, and provides an early indication of treatment efficacy in individual patients. Therefore, theranostic tests, which lead to rapid and more accurate diagnosis and allow for a more efficient use of drugs, and thus improved patient management, are increasingly used in cancer, cardiovascular and infectious diseases, or prediction of drug toxicity [79,80].

In summary, clinical bioinformatics has evolved into an essential tool in translational research, transforming fundamental bioinformatics research to clinical application by exploiting novel profiling technologies, biological databases, data mining and biostatistics methods for speeding up biomarker and drug discovery. These useful innovations will ultimately improve individualized clinical management of patient health and will also reduce costs of drug development.

## Abbreviations (in alphabetical order)

AFS: aggregating feature selection; Arg: arginine; AUC: area under the ROC curve; AV: associative voting; DA: discriminatory ability; GBA-FS: guild-by-association feature selection; GC: gas chromatography; GCP: good clinical practice; IG: information gain; IMR: ion-molecule reaction; IQR: interquartile range; KEGG: Kyoto Encyclopedia of Genes and Genomes; k-NN: k-nearest neighbor classifier; LC: liquid chromatography; LOCI: local correlation integral; LOF: local outlier factor; MCADD: medium-chain acyl-CoA dehydrogenase deficiency; MS: mass spectrometry; MS+: metabolic syndrome + cardiovascular disease; OMIM: Online Mendelian Inheritance in Man; Orn: ornithine; pBI: paired biomarker identifier; PCA: principal component analysis; Phe: phenylananine; PMI: planned myocardial infarction; PKU: phenylananine hydroxylase deficiency; PTR: proton transfer reaction; Q_1_: first quartile; Q_3_: third quartile; RD: redundancy demoting; RF: relief; RFM: random forest model; ROC: receiver operating characteristics; SFR: stacked feature ranking; SMI: spontaneous myocardial infarction; SVM-REF: support vector machine-recursive feature elimination; Thy: tyrosine; uBI: unpaired biomarker identifier.

## Competing interests

The authors declare that they have no competing interests.

## Authors' contributions

CB and DB conceptualized and wrote the manuscript. MO and MN prepared table and figures and commented on the paper. All authors have read and approved the final manuscript.
